# Mitochondrial reactive oxygen is critical for IL-12/IL-18-induced IFN-γ production by CD4^+^ T cells and is regulated by Fas/FasL signaling

**DOI:** 10.1038/s41419-022-04907-5

**Published:** 2022-06-06

**Authors:** Gorjana Rackov, Parinaz Tavakoli Zaniani, Sara Colomo del Pino, Rahman Shokri, Jorge Monserrat, Melchor Alvarez-Mon, Carlos Martinez-A, Dimitrios Balomenos

**Affiliations:** 1 Department of Immunology and Oncology, Centro Nacional de Biotecnología- Consejo Superior de Investigaciones Científicas (CNB-CSIC), Madrid, Spain; 2 Department of Medicine and Medical Specialties, University Alcalá de Henares, Alcalá de Henares, Spain; 3 Ramón y Cajal Institute of Sanitary Research (IRYCIS), Madrid, Spain; 4 Service of Internal Medicine and Immune System Diseases-Rheumatology, University Hospital Príncipe de Asturias, (CIBEREHD), Alcalá de Henares, Spain; 5 Present Address: BioMed X Institute (GmbH), Im Neuenheimer Feld 583, 69120 Heidelberg, Germany; 6 Present Address: Product and Research Complex, Pasteur Institute of Iran, Karaj, Iran

**Keywords:** Autoimmunity, Cytokines

## Abstract

Mitochondrial activation and the production of mitochondrial reactive oxygen species (mROS) are crucial for CD4^+^ T cell responses and have a role in naïve cell signaling after TCR activation. However, little is known about mROS role in TCR-independent signaling and in recall responses. Here, we found that mROS are required for IL-12 plus IL-18-driven production of IFN-γ, an essential cytokine for inflammatory and autoimmune disease development. Compared to TCR stimulation, which induced similar levels of mROS in naïve and memory-like cells, IL-12/IL-18 showed faster and augmented mROS production in memory-like cells. mROS inhibition significantly downregulated IFN-γ and CD44 expression, suggesting a direct mROS effect on memory-like T cell function. The mechanism that promotes IFN-γ production after IL-12/IL-18 challenge depended on the effect of mROS on optimal activation of downstream signaling pathways, leading to STAT4 and NF-κB activation. To relate our findings to IFN-γ-driven lupus-like disease, we used Fas-deficient memory-like CD4^+^ T cells from *lpr* mice. Importantly, we found significantly increased IFN-γ and mROS production in *lpr* compared with parental cells. Treatment of WT cells with FasL significantly reduced mROS production and the activation of signaling events leading to IFN-γ. Moreover, Fas deficiency was associated with increased mitochondrial levels of cytochrome C and caspase-3 compared with WT memory-like cells. mROS inhibition significantly reduced the population of disease-associated *lpr* CD44^hi^CD62L^lo^CD4^+^ T cells and their IFN-γ production. Overall, these findings uncovered a previously unidentified role of Fas/FasL interaction in regulating mROS production by memory-like T cells. This apoptosis-independent Fas activity might contribute to the accumulation of CD44^hi^CD62L^lo^CD4^+^ T cells that produce increased IFN-γ levels in *lpr* mice. Overall, our findings pinpoint mROS as central regulators of TCR-independent signaling, and support mROS pharmacological targeting to control aberrant immune responses in autoimmune-like disease.

## Introduction

Upon TCR activation, mitochondria rapidly translocate to the immunological synapse, leading to electron transport chain (ETC) activation [1] and mitochondrial reactive oxygen species (mROS) production. Superoxide (O_2_^—•^) is primarily generated at mitochondrial complexes I and III [2, 3], and it can reach cytosol either directly through mitochondrial membrane or after conversion to hydrogen peroxide (H_2_O_2_) [4]. It has recently been established that mROS play a crucial role as redox signaling molecules in T cells, regulating IL-2 and IL-4 production upon TCR triggering [5, 6].

IL-12 plus IL-18 (IL-12/IL-18) are required to boost T_H_1 responses and IFN-γ production after TCR ligation [7, 8]. IFN-γ can be induced by IL-12/IL-18 independently of TCR in a mechanism evolved to provide an early source of IFN-γ and contribute to the innate immune response [9, 10]. This “cytokine-induced cytokine production” could create a cycle of chronic inflammation [11–13]. We previously demonstrated that *lpr* T cell-produced IFN-γ amplifies macrophage-dependent inflammation [14].

Signaling mechanisms that drive IFN-γ production in response to IL-12/IL-18 differ from those downstream of TCR, and lead to differential NF-κB recruitment to *Ifng* locus [15–17]. Although the role of mROS has been established downstream of TCR, their role in IL-12/IL-18-dependent signaling remains unexplored. Excessive production of ROS is thought to underlie some of the pathological immune reactions implicated in autoimmunity. T cells from lupus patients exhibit mitochondrial hyperpolarization and increased mROS production [18, 19]. In a previous study, we demonstrated that autoimmune traits in *lpr* mice were decreased by controlling autoreactive T cell hyperactivation and their IFN-γ overproduction [14]. However, a direct link between mROS and IFN-γ hyperproduction in Fas-deficient T cells has not been made thus far.

Here, we hypothesized that mROS modulates signal transduction leading to hyperproduction of IFN-γ. We analyzed the role of mROS in IL-12/IL-18-induced IFN-γ production in naïve and in in vitro-differentiated memory-like CD4^+^ T cells, and found significantly higher amounts of mROS and IFN-γ in the later. We demonstrated that mROS signaling controls both STAT4 and NF-κB activation pathways. Finally, Fas-deficient CD4^+^ T cells produced more mROS compared to WT counterparts in response to IL-12/IL-18. This was translated to increased IFN-γ production by CD44^hi^CD62L^lo^ *lpr* CD4^+^ compared to WT T cells. Our findings establish mROS as key component of TCR-independent IFN-γ production and uncover an unknown role of Fas in regulating mROS and IFN-γ production.

## Results

### Enhanced mROS and IFN-γ responses of memory-like CD4^+^ T cells in response to IL-12/IL-18 challenge

**Fig. 1:**
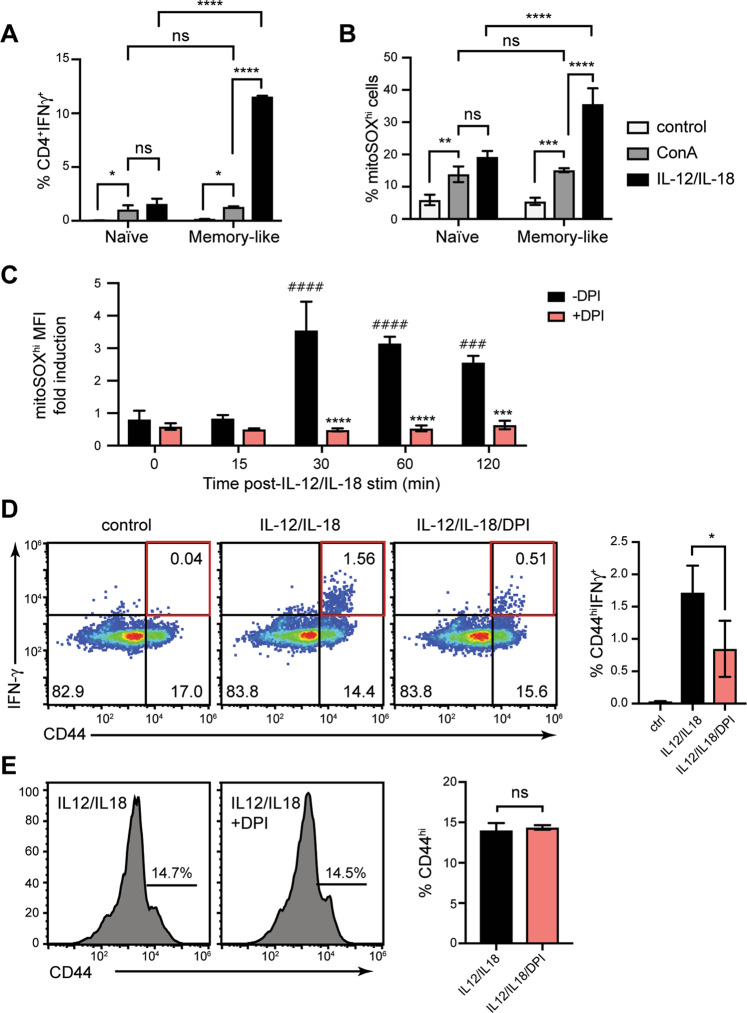
Memory-like CD4^+^ T cells exhibit increased mROS and IFN-γ production in response to IL-12/IL-18. Naïve CD4^+^ T cells were isolated from C57BL/6 mouse spleens and stimulated with ConA or IL-12/IL-18. To generate memory-like CD44^hi^ T cells, ConA-stimulated CD4^+^ cells were expanded in IL-2 for 6 days and re-stimulated with either ConA or IL-12/IL-18. **A** Percentage of IFN-γ-producing naïve and memory-like T cells within the CD4^+^ population, obtained by flow cytometry analysis 24 h after stimulation with ConA or IL-12/IL-18. **B** Percentage of mitoSOX^hi^ cells in naïve and memory-like cells 1 h after ConA or IL-12/IL-18 stimulation, obtained by flow cytometry analysis. **C** MitoSOX red fluorescence staining in mitoSOX^hi^ population of naïve CD4^+^ T cells at early time points after IL-12/IL-18 or IL-12/IL-18 + DPI treatment. Statistical significance is represented by symbols ^#^ for MFI increase after stimulation compared to the 0-time point, or * for MFI decrease after DPI treatment compared to untreated control of each time point **D** *Left*, Intracellular staining showing IFN-γ-producing CD44^hi^ cells after 24 h of Il-12/IL-18 or IL-12/IL-18 + DPI treatment of naïve CD4^+^ cells, evaluated by flow cytometry. *Right*, Statistical analysis of observed differences. **E** Surface expression of CD44 activation marker 24 h after IL-12/IL-18 or IL-12/IL-18 + DPI stimulation. Statistical significance was estimated using unpaired Student’s *t* test **E**, one-way ANOVA with post-hoc Tukey test (**D**) or two-way ANOVA with Sidak’s correction for multiple comparison (**A**, **B**, **C**). Histograms and pseudo-color plots are representative of at least three experiments performed. The graphs show mean ± SD (*n* = 3 different mice), *p < 0.05, **p < 0.01, ***p < 0.001, ****p < 0.0001.

Naïve CD4^+^ T cells purified from mouse spleens and characterized as CD44^lo^CD62L^hi^ (Supplementary Fig. 1) were stimulated ex vivo with IL-12/IL-18 to induce IFN-γ production independently of TCR, as previously described [20]. Alternatively, T cells were exposed to concanavalin A (ConA) that emulates the physiological TCR cross-linking leading to T cell activation and ROS production [21]. After primary TCR activation and IL-2 dependent culture, T cells efficiently differentiated into memory-like CD44^hi^ phenotype with both central (CD44^hi^CD62L^hi^) and effector (CD44^hi^CD62L^lo^) subsets (Supplementary Fig. 1), as previously described [22]. Memory-like CD4^+^ T cells were re-stimulated with either ConA or with IL-12/IL-18. TCR stimulation-induced low IFN-γ production in both naïve and memory-like cells (Fig. 1A). IL-12/IL-18 induced similar IFN-γ levels as ConA in naïve CD4^+^ cells, while in memory-like cells IFN-γ production was strikingly higher after IL-12/IL-18 stimulation (Fig. 1A).

Mitochondrial superoxide production was detected with two dyes, MitoSOX and MitoROS (see below). After ConA treatment, the percentage of MitoSOX^hi^ cells was increased compared to unstimulated cells, but we found no significant differences in mROS production between naïve and memory-like cells (Fig. 1B). In naïve cells, the proportions of MitoSox^hi^ cells after IL-12/IL-18 stimulation were similar to those induced by ConA (Fig. 1B). However, IL-12/IL-18 induced significantly higher proportions of MitoSOX^hi^ cells in the memory-like compared to naïve cells (Fig. 1B). Results were similar using MitoSOX MFI (median fluorescence intensity) (Supplementary Fig. 2).

Overall, memory-like cells displayed enhanced responses after IL-12/IL-18 compared to ConA stimulation in terms of both IFN-γ and mROS production, while these two types of stimulation had similar effects on naïve T cells.

### mROS controls IL-12/IL-18-dependent IFN-γ production upon naive CD4^+^ T cell activation

MitoSOX^hi^ cells significantly increased their MFI at 30- and 60-min post IL-12/IL-18 or ConA stimulation, while MitoSOX^lo^ cells were unresponsive (Supplementary Fig. 3 and data not shown). We thus inhibited mROS production to investigate whether mROS are functionally related to IL-12/IL-18-induced IFN-γ production in naïve CD4^+^ T cells.

Treatment with DPI (diphenyleneiodonium) [23, 24] suppressed mROS production in all time points tested (Fig. 1C). The proportion of IFN-γ-producing cells after IL-12/IL-18 stimulation (~1.5%) was significantly reduced by DPI treatment (Fig. 1D), whereas the overall proportion of CD44^hi^ cells was unaffected (Fig. 1E). Our results suggest that mROS controls IFN-γ production upon IL-12/IL-18 activation of naïve CD4^+^ T cells.

### mROS is critical for IFN-γ production by memory-like CD4^+^ T cells after IL-12/IL-18 challenge

**Fig. 2:**
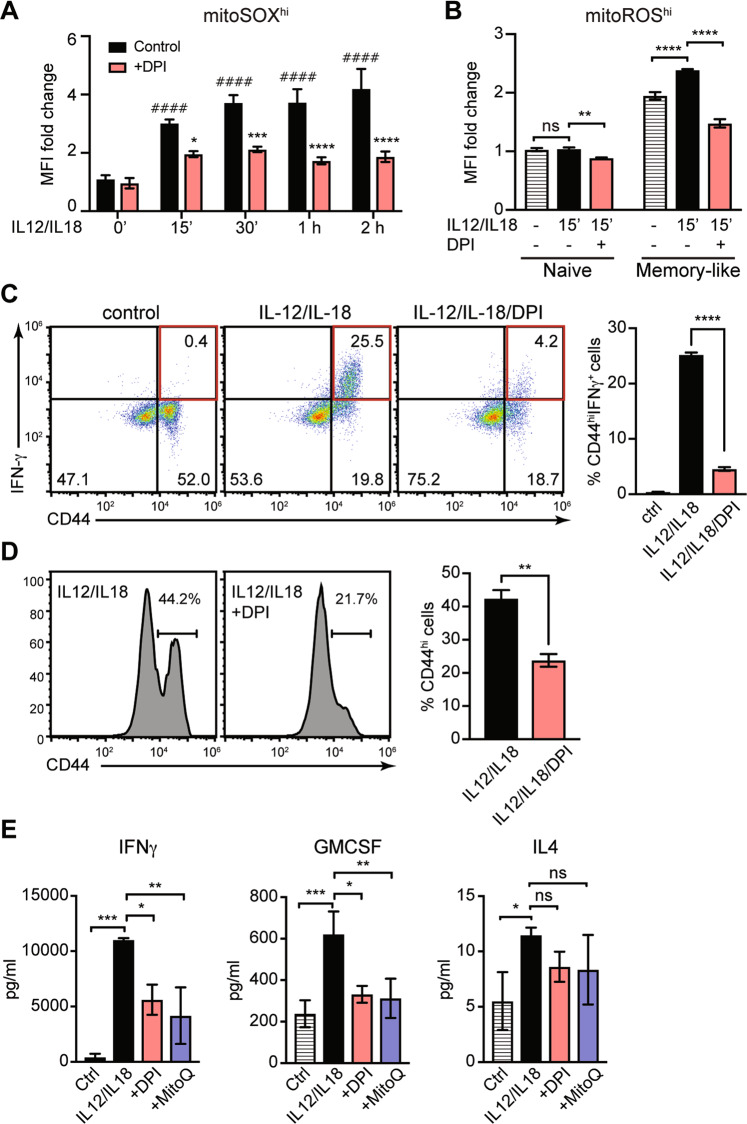
DPI treatment lowers mROS and IFN-γ production in differentiated memory-like CD4^+^ T cells. In vitro differentiated memory-like CD4^+^ T cells (as in Fig. 1) were stimulated with IL-12/IL-18. **A** MitoSOX red fluorescence analyzed by flow cytometry showing mROS production in the early time points of IL-12/IL-18 or IL-12/IL-18 + DPI treatment. Statistical significance is represented by symbols ^#^ for MFI increase after stimulation compared to 0 time point, or * for MFI decrease after DPI treatment compared to untreated control of each time point. **B** Flow cytometry analysis of mROS production using MitoROS dye in naïve *vs*. memory-like cells at 15 min after treatment with IL-12/IL-18 or IL-12/IL-18 + DPI. **C** *Left*, Intracellular staining showing the frequency of IFN-γ-producing CD44^hi^ effector/memory T cells at 24 h after IL-12/IL-18 or IL-12/IL-18 + DPI treatment in CD4^+^ cells. *Right*, Statistical analysis of observed differences. **D** Flow cytometry analysis showing surface expression of CD44 at 24 h after IL-12/IL-18 or IL-12/IL-18 + DPI treatment. **E** Cytokine secretion at 4 h after IL-12/IL-18 treatment in presence of DPI or MitoQ. Pseudo-color plots and histograms are representative of at least three experiments performed. Statistical significance was estimated using unpaired Student’s *t* test **D**, one-way ANOVA with post-hoc Tukey test (**C**, **E**) or two-way ANOVA with Sidak’s correction for multiple comparison **A**, **B**. Graphs show mean ± SD (*n* = 3 different mice); *p < 0.05; **p < 0.01; ***p < 0.001; ****p < 0.0001.

In contrast to the naïve cells, in which mROS was induced at 30 min after IL-12/IL-18 stimulation (Fig. 1C), in memory-like cells mROS was increased as early as 15 min post IL-12/IL-18 stimulation, as detected by MitoSOX and MitoRos (Fig. 2A, B). The impact of mROS on cytokine-induced signaling may thus be more relevant in memory-like than in naïve CD4^+^ T cells. DPI led to a prominent reduction of mROS at all time points tested after IL-12/IL-18 treatment (Fig. 2A, B). Because DPI under certain conditions might affect electron transport activity and mitochondrial membrane potential [25], we used the potentiometric TMRM staining to assess mitochondrial membrane potential and we found no significant changes after IL-12/IL-18 challenge with or without DPI (Supplementary Fig. 4).

Memory-like cells were highly responsive to IL-12/IL-18 stimulation, as ~25% of the total CD4^+^ population were CD44^hi^IFN-γ^+^ at 24 h post-stimulation (Fig. 2C). DPI treatment nearly abrogated IFN-γ production in memory-like T cells (Fig. 2C) without affecting cell viability or cell cycle (Supplementary Fig. 5). Moreover, DPI led to significant CD44 downregulation, suggesting that mROS regulates the activation status of these cells (Fig. 2D). Furthermore, treatment with mitochondrially targeted antioxidant MitoQ had similar effect as DPI in lowering IFN-γ and GM-CSF production induced by IL-12/IL-18 (Fig. 2E). In contrast, the induction of IL-4 production was very low, and it was not influenced by DPI or MitoQ (Fig. 2E). Our results suggest that mROS are vital for controlling IFN-γ production and the CD44^hi^ phenotype in IL-12/IL-18-stimulated memory-like T cells.

### Attenuation of IFN-γ production through different mROS inhibition approaches

**Fig. 3:**
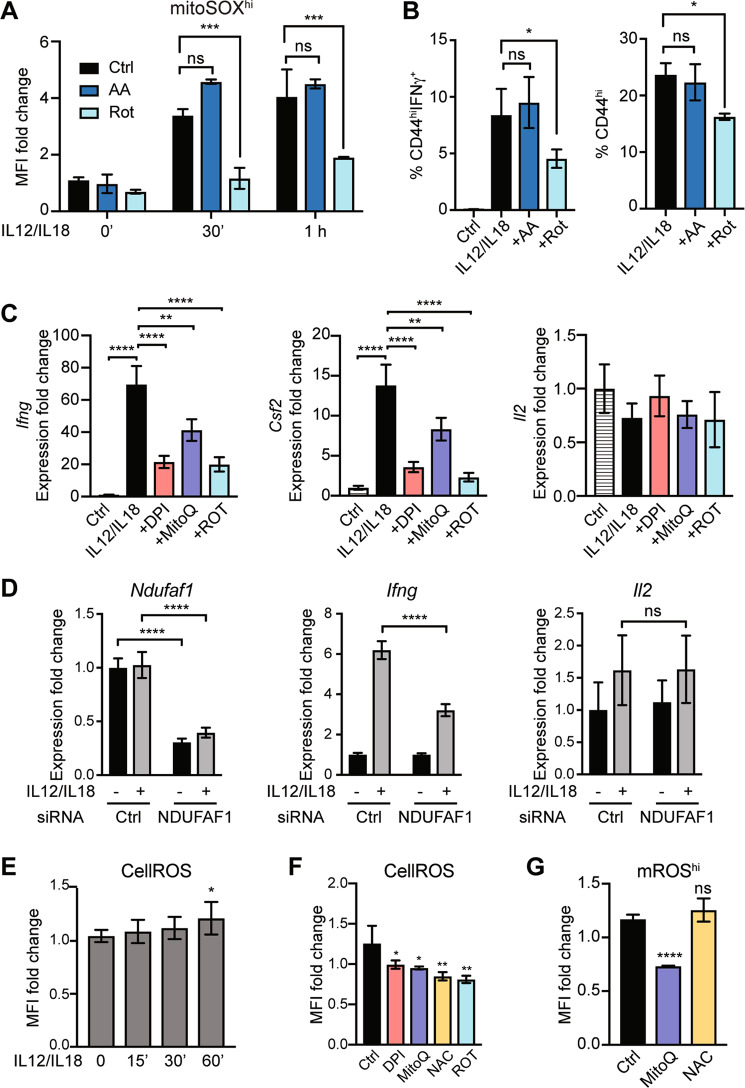
The effect of different inhibitors on IL-12/IL-18-triggered IFN-γ production in differentiated memory-like CD4^+^ T cells. In vitro differentiated memory-like CD4^+^ T cells (as in Fig. 1) were stimulated with IL-12/IL-18. **A** mROS production in mitoSOX^hi^ population was reduced by rotenone treatment but not by antimycin A. **B** Effect of rotenone and antimycin A treatment on IFN-γ-production and CD44 expression by CD4^+^ cells. Data were obtained by flow cytometry. **C** qRT-PCR analysis of IFN-γ, GM-CSF (Csf2) and IL-2 expression in memory-like cells at 1 h after treatment with IL-12/IL-18 or IL-12/IL-18 plus inhibitors (DPI, rotenone) or MitoQ. **D** qRT-PCR analysis of NDUFAF1, IFN-γ, and IL-12 expression in siCtrl or siNDUFAF1-transfected memory-like CD4^+^ T cells after treatment with IL-12/IL-18. **E** Flow cytometry analysis of cellular ROS production after IL-12/IL-18 treatment. Flow cytometry analysis of CellROS (**F**) and mROS **G** production in memory-like cells stimulated with IL-12/IL-18 (Ctrl) or IL-12/IL-18 plus inhibitors (DPI, rotenone) or antioxidants (MitoQ, NAC). The samples for qRT-PCR, cellROS and mROS measurement were normalized to unstimulated cells of each treatment condition. Graphs show mean ± SD (*n* = 3 different mice); *p < 0.05; **p < 0.01; ***p < 0.001; ****p < 0.0001; ns, not significant; two-way ANOVA with Sidak’s correction **A**, **D**; one-way ANOVA with post-hoc Tukey test **B**, **C**, **E**, **F**, **G**.

As DPI might also inhibit Nox enzymes [23, 26–28], we tested other respiratory chain blockers to verify the role of mROS in IFN-γ production. Specific complex I inhibitor rotenone significantly decreased mROS production (Fig. 3A) and the percentages of IFN-γ^+^ and CD44^hi^ cells induced by IL-12/IL-18 (Fig. 3B), while antimycin A (complex III inhibitor) had no such effect (Fig. 3A and B).

At 1 h post-IL-12/IL-18 stimulation we detected substantially increased mRNA levels of IFN-γ (>60-fold) and GM-CSF (>10-fold), while the mRNA of IL-2, a cytokine governed by TCR activation, remained unaffected (Fig. 3C). DPI, rotenone, and MitoQ significantly reduced IFN-γ and GM-CSF expression at 1h-post IL-12/IL-18 treatment, without affecting IL-2 mRNA (Fig. 3C).

To exclude unspecific effects of pharmacological inhibition, we used siRNA to downregulate NDUFAF1, an essential chaperone for mitochondrial complex I assembly [29]. NDUFAF1 siRNA-transfected cells displayed ~50% decrease in NDUFAF1 and IFN-γ expression compared with control siRNA (Fig. 3D). IL-2 was neither induced by IL-12/IL-18 nor affected by NDUFAF1 siRNA (Fig. 3D).

We next measured the cellular ROS (cellROS) levels using a general ROS indicator CM-H_2_DCFDA. CellROS were not significantly increased until 1 h post-IL-12/IL-18 stimulation (Fig. 3E). Treatment with mROS inhibitors DPI, MitoQ, and rotenone, or with intracellular antioxidant NAC significantly decreased cellROS levels (Fig. 3F). On the other hand, the levels of mROS were reduced by MitoQ but not by NAC (Fig. 3G). This suggested that mROS precedes and possibly accounts for cellROS through ROS leakage from mitochondria after IL-12/IL-18 stimulation.

Collectively, these data show that mitochondrial complex I and mROS are essential for IFN-γ production by memory-like CD4^+^ T cells after IL-12/IL-18 treatment.

### mROS-mediated signaling activates IFN-γ production through STAT4 and NF-κB

**Fig. 4:**
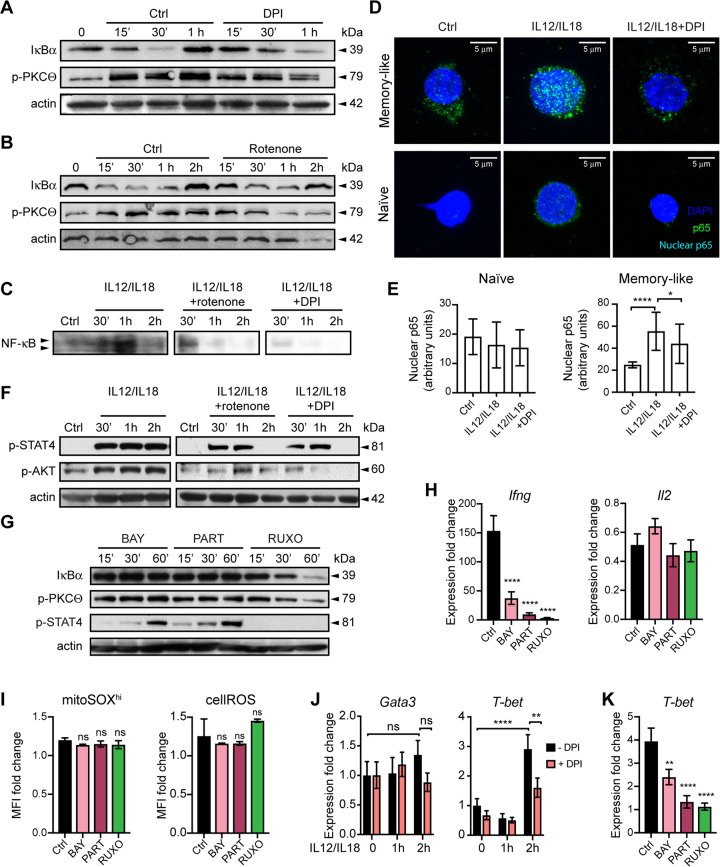
IL-12/IL-18 signaling activates IFN-γ production through mROS-dependent STAT4 and NF-κB. In vitro differentiated memory-like CD4^+^ T cells (as in Fig. 1) were stimulated with IL-12/IL-18. Immunoblot analysis showing IκBα and p-PKC-θ levels before and after DPI (**A**) or rotenone **B** treatment. **C** EMSA analysis showed reduced NF-κB nuclear activity after DPI and rotenone treatment. **D** Representative confocal microscopy images of memory-like cells stimulated with IL-12/IL-18 or IL-12/IL-18 + DPI for 15 min and stained with anti-p65 antibody and DAPI to visualize p65 nuclear translocation. The spot-like appearance of p65 is due to the low level of p65 translocation and/or the employed antibody. **E** Quantification of confocal microscopy images showing nuclear p65 abundance from three independent experiments and a minimum of 30 cells per condition. Protein phosphorylation after stimulation with IL-12/IL-18 or IL-12/IL-18 plus DPI or rotenone (**F**) or IL-12/IL-18 plus BAY, Parthenolide or Ruxolitinib **G** treatment. **H** qRT-PCR of IFN-γ and IL-2 expression at 1 h after stimulation with IL-12/IL-18 (Ctrl) or IL-12/IL-18 plus BAY, Parthenolide, or Ruxolitinib treatment. **I** Flow cytometry analysis of mROS and cellROS levels at 1 h after stimulation with IL-12/IL-18 (Ctrl) or IL-12/IL-18 plus BAY, Parthenolide, or Ruxolitinib treatment. **J, K** qRT-PCR analysis of T-bet and Gata3 expression after stimulation with IL-12/IL-18 (Ctrl) or IL-12/IL-18 plus BAY, Parthenolide or Ruxolitinib treatment. For **A**, **B**, **F**, and **G**, representative gels of at least two experiments are shown and β-actin was used as loading control. The samples for qRT-PCR, cellROS, and mROS measurement were normalized to unstimulated cells of each treatment condition. Graphs show mean ± SD (*n* = 3 different mice); *p < 0.05; **p < 0.01; ***p < 0.001; ****p < 0.0001; one-way ANOVA with post-hoc Tukey test.

IL-12 and IL-18 act in synergy to activate IFN-γ production through STAT4 and NF-κB, respectively [20, 30–32]. At 0 time point NF-κB was kept in latent state, as seen by high protein levels of IκBα (Fig. 4A and B; for all western blots original data are provided in supplementary material). As early as 15 min after IL-12/IL-18 stimulation IκBα levels dropped, which was halted after DPI and rotenone, suggesting that early mitochondrial oxidative signal after IL-12/IL-18 signaling promotes NF-κB activation (Fig. 4A, B). Electromobility shift assay showed elevated NF-κB nuclear activity at 1 h post-IL-12/IL-18 treatment and its marked decrease by DPI or rotenone (Fig. 4C). Furthermore, confocal microscopy indicated increased nuclear accumulation of p65 NF-κB at 15 min after IL-12/IL-18 treatment in memory-like cells, which was not evident in naïve cells and it was markedly decreased after DPI treatment (Fig. 4D). These data were statistically significant, following quantification of the nuclear p65 translocation (Fig. 4E).

Phosphorylation of STAT4 was evident after IL-12/IL-18 stimulation and was markedly decreased by both rotenone and DPI (Fig. 4F), suggesting a direct mROS role in IL-12-dependent STAT4 activation. We also found increased PKC-θ and AKT phosphorylation after IL-12/IL-18 treatment that was prevented by both mROS inhibitors (Fig. 4 A, B, and F).

Next, we used pharmacological inhibition to confirm the role of NF-κB and STAT4 in mROS-driven IFN-γ production induced by IL-12/IL-18. NF-κB inhibitors Bay and Parthenolide (Part) [33] prevented IκBα degradation, while the Janus kinase inhibitor Ruxolitinib (Ruxo) [34] suppressed STAT4 and PKC-θ phosphorylation upon IL-12/IL-18 stimulation (Fig. 4G). All three inhibitors severely impaired the induction of IFN-γ expression, while IL-2 was not affected (Fig. 4H), confirming the role of NF-κB and STAT4 in IL-12/IL-18-induced IFN-γ production. Last, mROS and cellROS production were not affected by any of the three inhibitors (Fig. 4I), demonstrating that mROS regulate NF-κB and STAT4, and not vice versa, at least until 1h-post IL-12/IL-18 stimulation.

In view of the critical role of T-bet in regulating IFN-γ production in Th1 cells [35], we observed that T-bet mRNA levels increased at 2h-post IL-12/IL-18 stimulation, while Gata3 mRNA remained unchanged (Fig. 4J). This indicated that T-bet is likely required for sustained IFN-γ production at later time points of IL-12/IL-18 stimulation. T-bet increase was inhibited by DPI and by NF-κB and STAT4 inhibitors (Fig. 4J, K).

**Fig. 5:**
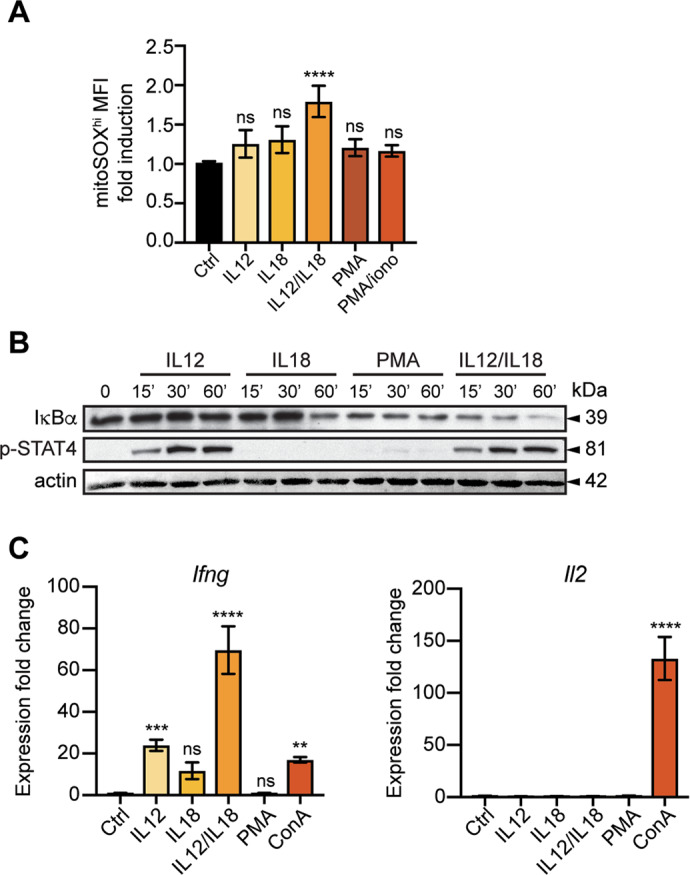
IL-12 and IL-18 act in synergy to induce mROS and IFN-γ production. In vitro differentiated memory-like CD4^+^ T cells (as in Fig. 1) were stimulated as indicated. **A** Flow cytometry analysis of mitoSOX^hi^ population at 60 min after stimulation with different stimuli. **B** Immunoblot analysis at early time points after treatment with IL-12 alone, IL-18 alone, PMA, or IL-12/IL-18. Shown is representative gel of two experiments performed. β-actin was used as loading control. **C** qRT-PCR analysis of IFN-γ and IL-2 expression at 1 h after stimulation with different stimuli. All samples were normalized to unstimulated (Ctrl) cells. Graphs show mean ± SD (*n* = 3 different mice); ****p < 0.0001; ns not significant; one-way ANOVA with post-hoc Tukey test.

We next assessed the individual roles of IL-12 and IL-18 in mROS production. We observed that IL-12 and IL-18 act in synergy to induce mROS production, as IL-12 or IL-18 alone did not significantly increase mROS levels compared with untreated cells (Fig. 5A). We also tested PMA and PMA/ionomycin, and we found no significant mROS production compared with untreated cells (Fig. 5A).

In agreement with this, IL-12 alone did not induce IκBα degradation while IL-18 did, although to a much lesser extent than PMA or IL-12/IL-18 (Fig. 5B). On the other hand, IL-12 increased STAT4 phosphorylation (Fig. 5B). Accordingly, IFN-γ expression required synergy between IL-12/IL-18 as single IL-12 and IL-18 showed much lower IFN-γ induction, comparable to that of ConA (Fig. 5C). IL-2 was not induced by IL-12 or IL-18 as already shown (Fig. 3C and G), while ConA induced high IL-2 production (Fig. 5C).

Altogether, these data further strengthen the notion that mROS trigger early signaling events downstream of IL-12R and IL-18R leading to IFN-γ production.

### Fas deficiency results in mROS and IFN-γ overproduction and CD44^hi^CD62L^lo^ CD4^+^ T cell accumulation

**Fig. 6:**
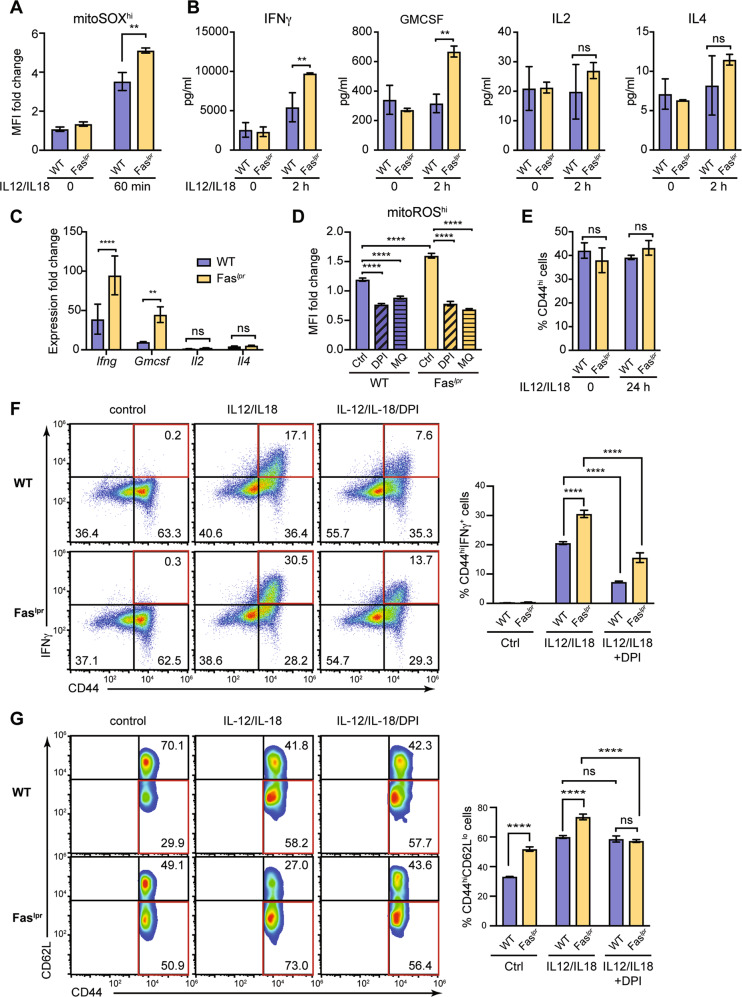
Increased IFN-γ production by *lpr* CD44^hi^ T cells depends on mROS production. In vitro differentiated memory-like CD4^+^ T cells from C57BL/6 (WT) and C57BL/6-*lpr* mice (Fas^*lpr*^) mice were stimulated with IL-12/IL-18. **A** Flow cytometry analysis of mitoSOX^hi^ population at 60 min after stimulation with IL-12/IL-18. **B** Secretion of cytokines at 2 h and **C** qRT-PCR analysis of cytokine expression at 4 h after IL-12/IL-18 stimulation. Fold change over unstimulated. **D** Flow cytometry analysis of mitoROS^hi^ population at 15 min after treatment with IL-12/IL-18 or IL-12/IL-18 plus DPI or MitoQ. The samples were normalized to unstimulated cells of each treatment condition. **E** CD44 surface expression at 24 h after IL-12/IL-18 treatment, analyzed by flow cytometry. **F** *Left*, Intracellular staining showing the frequency of IFN-γ-producing CD44^hi^ cells 24 h after IL-12/IL-18 or IL-12/IL-18 + DPI treatment. *Right*, Statistical analysis of observed differences. **G** *Left*, Flow cytometry analysis of CD62L and CD44 surface expression within the CD4^**+**^CD44^hi^ population at 24 h after IL-12/IL-18 or IL-12/IL-18 + DPI treatment. *Right*, Statistical analysis of observed differences. Pseudo-color plots are representative of three experiments performed. Graphs show mean ± SD (*n* = 3 different mice); *p < 0.05; **p < 0.01; ***p < 0.001; ****p < 0.0001; ns not significant; two-way ANOVA with Sidak’s correction.

*lpr* (lymphoproliferation spontaneous mutation) mice are Fas (CD95) deficient and show defective activation-induced cell death of in vitro TCR re-stimulated T cells [36, 37]. Double negative (TCRαβ^**+**^CD4^**-**^CD8^**-**^B220^**+**^) T cells comprise the primary accumulating population in *lpr* mice. Memory T cells, including effector/memory CD44^hi^CD62L^lo^CD4^+^ T cells, also accumulate in these mice and hyperproduce IFN-γ^14^. The mechanism that drives this IFN-γ hyperproduction in the absence of Fas remains undefined. To investigate the role of mROS in the responses of Fas-deficient memory-like cells, we purified CD4^+^ T cells from C57BL/6 (WT) and C57BL/6-*lpr* (*lpr*) mice and, after initial ConA stimulation and IL-2 expansion, we stimulated the cells with IL-12/IL-18. Remarkably, we detected significantly higher mROS levels in *lpr* compared to WT cells, as seen by MitoSOX at 60 min after stimulation (Fig. 6A). This was paralleled by significantly higher levels of IFN-γ and GM-CSF in *lpr* compared to WT cells, while IL-2 and IL-4 were not induced by IL-12/IL-18 (Fig. 6B, C). mROS production was greatly diminished by DPI and MitoQ in both WT and *lpr* cells (Fig. 6D). Similar data were obtained using disease-developing Fas-deficient mice on MRL/MpJ background (Supplementary Fig. 6). Cell cycle analysis showed no disparity in apoptosis or proliferation between WT and *lpr* CD4^+^ T cells after IL-12/IL-18 stimulation (Supplementary Fig. 7), suggesting that the observed differences were independent of the pro-apoptotic Fas role.

Although CD44^hi^ cell percentages were similar in WT and *lpr* cultures (Fig. 6E), the proportions of *lpr* CD44^hi^IFN-γ^+^ cells were significantly increased compared to WT (~17 vs. 30% of the total CD4^+^ population) at 24 h post-IL-12/IL-18 challenge (Fig. 6F). DPI significantly reduced CD44^hi^IFN-γ^+^ cells in both WT and *lpr* (Fig. 6F), indicating that increased mROS drive elevated IFN-γ production in *lpr* CD44^hi^ T cells.

In addition, CD44^hi^/CD62L^lo^ effector/memory cell proportions were increased in *lpr* compared to their WT counterparts before (29.9 vs. 50.9%, Fig. 6G) and after (~58.2 vs. 73%, Fig. 6G) IL-12/IL-18 stimulation. DPI did not affect proportions of effector/memory CD44^hi^CD62L^lo^ in WT cells, but it significantly reduced this population in *lpr* cultures (Fig. 6G). Altogether, our results corroborate that increased mROS production drives not only the hyperactivation and increased potential for IFN-γ production in Fas-deficient CD4^+^ T cells, but also their intensified differentiation towards the effector/memory CD44^hi^/CD62L^lo^ phenotype. These findings point to Fas as a negative regulator of mitochondrial ROS that independently of its proapoptotic role controls the activity of CD44^hi^ T cells.

### Fas/FasL interaction controls mROS and IFN-γ production after IL-12/IL-18 challenge

**Fig. 7:**
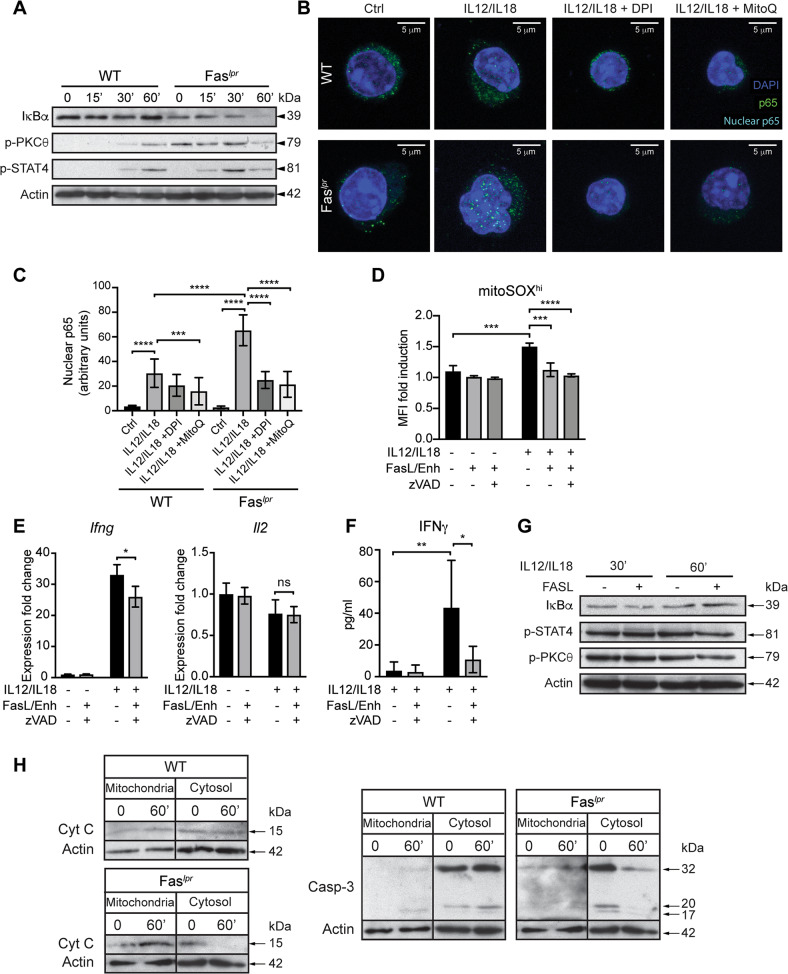
Fas/FasL interaction restricts mROS and IFN-γ production in memory-like CD4^+^ T cells. **A** Immunoblot analysis of memory-like CD4^+^ T cells from C57BL/6 (WT) and C57BL/6-*lpr* mice (Fas^*lpr*^) mice stimulated with IL-12/IL-18. **B** Representative confocal microscopy images of memory-like CD4^+^ T cells from WT and Fas^*lpr*^ mice stimulated with IL-12/IL-18 or IL-12/IL-18 + DPI or MitoQ for 15 min and stained with anti-p65 antibody and DAPI to visualize p65 nuclear translocation. **C** Quantification of confocal microscopy images showing nuclear p65 abundance from two independent experiments and a minimum of 20 cells per condition. **D** Flow cytometry analysis of WT mitoSOX^hi^ population at 30 min after IL-12/IL-18 stimulation in presence of soluble FasL and zVAD. **E** qRT-PCR analysis of IFN-γ and IL-2 (1 h after stimulation), **F** IFN-γ protein levels and **G** immunoblot analysis of WT cells after IL-12/IL-18 stimulation in presence of soluble FasL and zVAD. **H** Immunoblot analysis of mitochondrial and cytosol fraction in WT and Fas^*lpr*^ memory-like CD4^+^ T cells stimulated with IL-12/IL-18 for 1 h. For **A**, **G**, and **H**, representative gels of two experiments are shown and β-actin was used as loading control. Graphs show mean ± SD (*n* = 3 different mice); *p < 0.05; **p < 0.01; ***p < 0.001; ****p < 0.0001; two-way ANOVA with Sidak’s correction.

The mechanism underlying elevated mROS and IFN-γ production in Fas-deficient memory-like CD4 T cells involved markedly potentiated IκBα degradation and faster kinetics of PKC-θ and STAT4 phosphorylation compared with WT cells (Fig. 7A). Confocal microscopy data and data quantification revealed significantly increased p65 nuclear translocation in Fas-deficient T cells compared to WT shortly after IL-12/IL-18 stimulation, which was decreased after DPI or MitoQ treatment (Fig. 7B, C). This suggested that the increased mROS production results in elevated NF-κB activity in *lpr* compared to WT T cells.

To next assess the role of Fas/FasL signaling in IL-12/IL-18-induced mROS production, we treated WT cells with soluble FasL in presence of cross-linking enhancer, and found significantly decreased mROS production after 1 h of treatment (Fig. 7D). Apoptosis induced by FasL was abrogated using z-VAD-fmk [38] (Supplementary Fig. 8). FasL treatment reduced IFN-γ production (both protein and mRNA), while IL-2 expression was unaffected (Fig. 7E, F). Mechanistically, FasL treatment reduced IκBα degradation and phosphorylation of STAT4 and PKC-θ (Fig. 7G). This suggests that Fas/FasL interaction directly controls mROS production and the pathway leading to IFN-γ production.

**Fig. 8:**
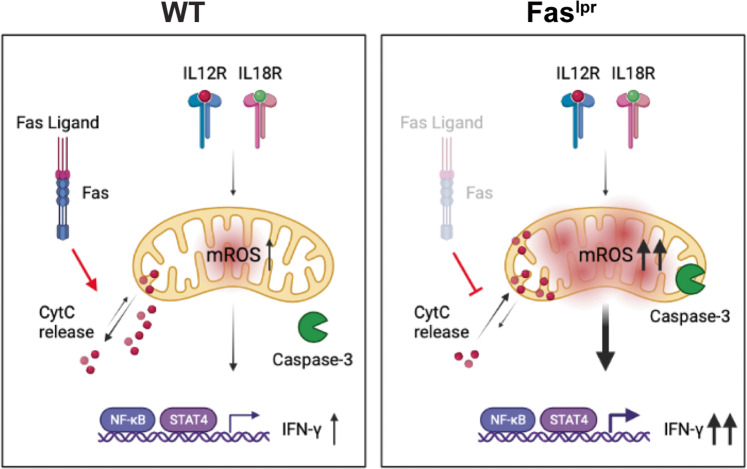
Model for Fas/FasL regulating mROS and IFN-γ production after IL-12/IL-18 stimulation. *Left*, IL-12/IL-18 stimulation triggers mitochondria activation and mROS production, which activate the pathway leading to IFN-γ expression. Fas/FasL interaction leads to cytochrome C release, which controls mitochondrial activity and mROS production [39] and, consequently, IFN-γ. Caspase-3 is located outside of mitochondria [58], but there is no apoptosis induction in response to IL-12/IL-18. *Right*, In absence of Fas, cytochrome C is retained in mitochondria and mROS production is increased. Fas deficiency provokes caspase-3 translocation to mitochondria after IL-12/IL-18 stimulation, which might further increase mROS production [54]. Overall, increased mROS production results in IFN-γ hyperproduction in absence of Fas/FasL interaction.

Mitochondrial cytochrome C functions as an electron shuttle in the respiratory chain. Following Fas/FasL interaction cytochrome C gets released from mitochondria, which may influence mROS [39]. Additionally, caspase-3 activity within mitochondria has been associated with increased mROS production [40]. Thus, we examined whether the absence of Fas affects the mitochondrial abundance of cytochrome C and Caspase-3 in a mitochondrial fractionation experiment. During the IL-2 culture mitochondrial cytochrome C levels were low with a substantial cytosol release in both WT and *lpr* T cells (0 time point Fig. 7H, left). This corroborates previous findings showing that IL-2 sensitizes mitochondria to apoptotic stimuli by releasing cytochrome C in presence of Fas/FasL [41]. In agreement with the fact that caspase-3 is mostly found outside mitochondria in IL-2 cultured cells [42], we observed high levels of caspase-3 in cytosol fraction in both WT and *lpr* T cells (Fig. 7H, right). Following IL-12/IL-18 stimulation, Fas-deficient memory-like cells showed markedly decreased cytosolic cytochrome C and caspase-3 levels and an increase in the mitochondrial fraction (Fig. 7H). These results support a model whereby Fas/FasL interaction promotes cytochrome C release from the mitochondria to the cytoplasm that might ultimately regulate mROS and IFN-γ production (Fig. 8).

## Discussion

TCR engagement induces mitochondrial activation and production of mROS that modulate the redox state of signaling molecules governing the early events downstream of TCR. Here, we analyzed the role of mROS in TCR-independent responses of naïve and memory-like CD4^+^ T cells. Although after TCR stimulation mROS induction was similar in naïve and memory-like cells, in response to IL-12/IL-18 memory-like cells showed much higher increase of mROS and IFN-γ production compared to naïve cells. This suggested that upon by IL-12/IL-18 stimulation memory-like CD4^+^ T cells acquire a singular dependence on mROS and the study was geared toward the analysis of mROS effects under such activation conditions. We report the following major findings: First, increased mROS production induced by IL-12/IL-18 in memory-like CD4^+^ T cells was directly associated to IFN-γ production and CD44^hi^ phenotype. Second, the mechanism that potentiates IFN-γ production is regulated by the effect of mROS on both IL-12R and IL-18R signaling pathways. Third, our results uncovered a previously unknown role of Fas/FasL in regulating mROS and IFN-γ production, which could be associated with mitochondrial cytochrome C and caspase-3 accumulation in *lpr* memory-like CD4^+^ cells.

TCR stimulation of naïve CD4^+^ cells triggers mROS, which act as secondary messengers regulating IL-2 production and antigen-specific proliferation [6, 43]. At later stages of TCR stimulation mROS production persists in parallel with glycolysis, suggesting a continuing role for mROS in T cell activation [44]. Although mitochondria is associated with enhanced TCR-dependent activation of memory CD8^+^ T cells [45], the role of mROS in recall responses has not been established. Cytosolic ROS is increased in in vitro memory-like CD4^+^ T cells after TCR-dependent stimulation, suggesting that it emanates from mROS production [5]. Here, we found similar mROS levels in naïve and memory-like CD4^+^ T cells early after TCR activation.

We established that mROS levels were linked to T cell hyperactivity in response to IL-12/IL-18 induction, as mROS inhibition greatly lowered IFN-γ and CD44 expression. mROS production has been correlated with changes in mitochondrial membrane potential [46]. In our system mitochondrial membrane potential remained unaffected by mROS inhibition, indicating that mROS mediate memory T cell hyperactivation independently of the membrane potential that is linked to ATP production.

Synergy between IL-12R and IL-18R signaling pathways [32] induces IFN-γ production. mROS inhibition reduced IL-12R-dependent STAT4 phosphorylation and IL-18-dependent NF-κB activation, indicating that mROS drive signaling of both pathways. Kinetic analysis after IL-12/IL-18 treatment, showed an early effect on mROS. Other molecules, such as PKC-θ or AKT linked to these pathways [47, 48], also showed reduced phosphorylation after mROS inhibition. Although PKC-θ activation has been implicated in TCR-independent NF-κB regulation [49], our data showed reduced PKC-θ activation after treatment with Ruxolitinib (Fig. 4G), suggesting a cross-talk between IL-12-driven STAT4 and PKC-θ in this system.

The redox conditions associated to increased mROS production could alter the phosphorylation patterns, as kinase and phosphatase activities are redox-dependent [50]. Whether increased mROS activate specific pathway components or exert a generalized effect on phosphorylation remains to be explored. Another important issue is to understand how mROS is activated after secondary IL-12/IL-18 stimulation. We hypothesize that there is a cross-talk between the components of IL-12R and/or IL-18R signaling pathways and ETC. Indeed, PKC-θ has been shown to interact with and activate mitochondria in other systems [51].

Compared to WT, *lpr* memory-like CD4^+^ T cells showed increased mROS production, NF-κB activation, and IFN-γ levels after IL-12/IL-18 activation. Furthermore, soluble FasL treatment of WT cells significantly impaired mROS and IFN-γ production, suggesting that Fas/FasL interaction directly controls NF-κB activation independently of apoptosis. Although the prevention of NF-κB activation is considered key for Fas/FasL-driven activation-induced apoptosis, the role of Fas/FasL in controlling NF-κB is still described as controversial [52]. Indeed, lack of Fas augments NF-κB activation and inflammatory cytokine secretion in a tumor cell system [53]. IL-12/1L-18 stimulation is an apoptosis-resistant process that activates NF-κB. In such conditions, increased signaling by soluble FasL addition could affect mitochondria and mROS production and lead to NF-κB inhibition.

To shed light on the mechanism that controls mROS through Fas/FasL we examined mitochondrial levels of cytochrome C and caspase-3, since cytochrome C leakage from mitochondria has been associated with mROS regulation [39]. Cytochrome C has been shown to induce caspase-3 activity within mitochondria, leading to mitochondrial dysfunction and increased mROS production [54]. Our data showed that in absence of Fas both cytochrome C and caspase-3 are markedly decreased in the cytoplasm and increased within mitochondria (Fig. 7H). These findings suggest a role for the Fas/FasL system in controlling mROS production through mitochondrial cytochrome C release after IL-12/IL-18 stimulation of memory-like T cells (Fig. 8). Further studies are clearly needed for the better understanding of the mechanism that would explain the effect of Fas on mROS regulation.

Overall, the results unveil a previously unknown apoptosis-independent role of Fas in regulating mROS production and associated hyperinflammatory outcomes. Our findings suggest mROS as promoters of inflammation in *lpr* mice and potentially other autoimmune disorders. In fact, mitochondrial dysfunction, as well as increased mROS and IFN-γ production characterize T cells in SLE patients and in lupus-prone mouse models [55, 56]. Therefore, blockade of mROS might provide a therapeutic strategy to decrease inflammation in T cell-mediated inflammatory disorders.

## Materials and methods

### Mouse strains

Mice (8–12 weeks old female) were used for CD4^+^ T cell isolation. C57BL/6 mice (WT) were from Harlan Interfauna Ibérica; Fas-deficient C57BL/6-*lpr* mice (Fas^*lpr*^), as well as MRL/MpJ and MRL/MpJ-Fas^*lpr*^, were from Jackson laboratories. Mice were kept in SPF conditions. All animal experiments were designed in compliance with European Union and national regulations, and were approved by the CNB Bioethics Committee and the Community of Madrid (10/149633.9/18).

### In vitro CD4^+^ T cell activation and memory-like differentiation

Naïve CD4^+^ T cells were purified from mouse spleens using Untouched Mouse CD4 Cells Dynabeads (Invitrogen). Cell purity was >95%, as measured by flow cytometry using anti-CD4-FITC, anti-CD8-PE, and anti-B220-APC (Beckman Coulter) antibodies. Cells were cultured in RPMI 1640 containing 50 U/ml penicillin, 100 μg/ml streptomycin, 50 μM 2-mercaptoethanol, 2 mM L-glutamine, 0.1 mM nonessential amino acids, 10 mM HEPES, 1 mM sodium pyruvate, 10% Fetal bovine serum. Naïve CD4^+^ T cells (10^6^/ml) were stimulated with concanavalin A (ConA; 1.5 μg/ml, Sigma) or with IL-12 (10 ng/ml, Sigma) plus IL-18 (10 ng/ml, Sigma) for indicated time points. For memory-like differentiation, naïve cells were stimulated with ConA (1.5 μg/ml) for 24 h, washed, and cultured (0.5 × 10^6^/ml) in presence of 20 ng/ml human recombinant interleukin-2 (rIL-2, PeproTech) for 6 days. For naïve and effector/memory phenotyping, cells were stained with anti-CD4-PeCy7 (GK1.5; BioLegend), anti-CD44-APC, and anti-CD62L-PE (11-7311-82; eBioscience). Differentiated memory-like cells were stimulated with ConA or with IL-12 (10 ng/ml) plus IL-18 (10 ng/ml) for indicated time points. Cells were exposed to DPI (5 μM; Sigma), rotenone (2.5 μM; Sigma), mitoquinone (MitoQ, 1 μM; MedChemExpress), antimycin A (4 μM; Sigma), N-acetyl-L-cysteine (NAC, 2 mM; Sigma), BAY 11-7082 (10 μM, Sigma), Parthenolide (10 μM, Sigma) or Ruxolitinib (1 μM, a kind gift from Estanislao Nistal-Villán at CEU San Pablo University, Madrid, Spain) for 30 min before the stimulus. FasL treatment (500 ng/ml; ALX-522-001, Enzo Life Sciences) was initiated 15 min before the stimulus, in presence of cross-linking enhancer (1 μg/ml; ALX-804-034, Enzo Life Sciences) and z-VAD-Fmk (50 μM; Bachem), as indicated.

### siRNA transfection

For transfection we used universal scrambled negative control (SR30004) and 3 siRNA duplexes specific for mouse Ndufaf1 (SR4 10958 A, B, and C, Supplementary Table 1), all from OriGene. Memory-like CD4^+^ T cells were transfected with 250 nM siRNA oligonucleotides using AMAXA Mouse T cell Nucleofector Kit (Lonza) according to the manufacturer’s instructions. After overnight recovery cells were subjected to the experimental procedures.

### Flow cytometry

For detection of mitochondrial superoxide, cells were incubated with MitoSOX Red (Invitrogen) or with MitoROS (Cayman Chemical) at 5 μM final concentration for 30 min at 37 °C and stimulated for indicated time points. Cells were then washed with PBS, centrifuged at 300 × *g* for 5 min, and analyzed immediately using flow cytometry. The fluorescence was detected at the excitation and emission wavelengths of 488 and 585 nm, respectively. As a general ROS indicator, we used CM-H_2_DCFDA (Invitrogen) at 5 μM final concentration, detected at 533 nm. To control for baseline fluorescence, untreated samples from each experiment were stained according to the above procedure. MFI (median fluorescence intensity) fold induction was calculated by dividing values of stimulated by unstimulated cells.

For measurement of mitochondrial membrane potential TMRM (Tetramethylrhodamine, Methyl Ester, Perchlorate; ThermoFisher) was added to cell cultures at a concentration of 30 nM. After 30 min, cells were analyzed by flow cytometry. Control cells were pre-incubated with 5 μM FCCP for 10 min at 37 °C to depolarize the membrane and show background staining.

To analyze cell cycle, 10^6^ cells were permeabilized with detergent and stained with PI according to the manufacturer’s instructions (DNA-Prep Reagent Kit, Beckman Coulter) for 30 min at 37 °C.

To quantify apoptosis, cells were stained using Annexin V-FITC (PharMingen; 1 μg/ml, 15 min in binding buffer). Propidium iodide (10 μg/ml) was added to distinguish live (Annexin V-negative and PI-negative) from early apoptotic (Annexin V-positive and PI-negative) and late apoptotic/necrotic (Annexin V-positive and PI-positive) cells.

To measure intracellular levels of IFN-γ, cells were stimulated for the indicated time and Brefeldin A (BioLegend) was added to cell culture during the last 3 h of stimulation. Cells were stained with violet LIVE/DEAD stain kit (Invitrogen) for dead cell exclusion. After surface marker staining, cells were fixed and permeabilized with Citofix/Citoperm kit (BD Biosciences) and blocked with of Fc-receptor-binding antibody (082732121, Beckman Coulter) for 15 min at 4 °C. Cells were stained intracellularly with anti-IFN-γ-FITC (1/100 dilution, eBioscience) and analyzed on a Gallios cytometer (Beckman Coulter). The data were analyzed using FlowJo software (Tree Star).

### Quantification of cytokines from culture supernatants

Cell culture supernatants were analyzed using Milliplex MAP mouse Cytokine Magnetic Bead Panel (Merck) as per manufacturer’s protocol. The plate was read in a Luminex MAGPIX with xPONENT software. Cytokine concentrations were calculated with Analyst software (Merck) and shown as absolute values in pg/ml.

### Quantitative polymerase chain reaction

To measure relative gene expression by qRT-PCR, total cellular RNA was extracted using TRI reagent (Sigma). Complementary (cDNA) was synthesized from 1 μg of RNA using Reverse Transcription Kit from Applied Biosystems. Quantitative PCR was then performed using EvaGreen Master Mix (Solis BioDyne), detected by the QuantStudio 5 and analyzed using QuantStudio Software v1.5.1 (Applied Biosystems). Results were normalized to the expression of β-actin and presented as fold induction with respect to the control cell population according to the 2^-ΔΔCT^ method. When mRNA levels were affected by treatment conditions in unstimulated cells, results were normalized to unstimulated cells of each condition to calculate the amplitude of the transcription response (“dynamic range”) after stimulation. All primer sequences used for qRT-PCR can be found in Supplementary Table 2.

### Immunoblotting

Cells were lysed with 0.2% NP-40 lysis buffer containing 10 mM Tris-HCl [pH 7.5], 150 mM NaCl, and 1 × protease and phosphatase inhibitor cocktail (Roche), and then centrifuged at 4 °C to obtain cellular lysate. Mitochondrial extracts were separated from cytoplasmic fraction using Mitochondria Isolation Kit (Thermo Scientific). Protein concentration was determined using Bio-Rad protein assay, equal amounts of protein (20–40 μg) were resolved in 12% SDS-PAGE and transferred to 0.2 μm nitrocellulose membranes. Membranes were incubated with primary antibodies at 4 °C overnight in 1 × TBS/0.05% Tween containing 5% bovine serum albumin, and then in horseradish peroxidase (HRP)-conjugated secondary antibodies (from Dako) at room temperature for 1 h and then developed with Western Lightning Plus-ECL (Perkin Elmer). The following primary antibodies were used: anti-p-STAT4 (sc-101804) and anti-Caspase 3 (sc-7148) from Santa Cruz Biotechnology; anti-p-AKT (#3787), anti-p-PKC-θ (#9377) and anti-IκBα (#9242) from Cell Signaling; cytochrome C (556433) from BD Biosciences and actin (AC-15) from Sigma.

### EMSA

Cells were washed with ice-cold PBS and nuclear extracts were obtained using nuclear/cytosol Fractionation kit (BioVision). Double-stranded NF-κB consensus oligonucleotides (5ʹ-AGT TGA GGG GAC TTT CCC AGG C-3ʹ from Promega) were end-labeled with [γ^32^P]ATP (PerkinElmer) using T4 polynucleotide kinase (Promega). In a 25 μl reaction volume, 5 μg nuclear extract was incubated with 0.5 ng labeled oligonucleotide probe, 10 mM HEPES, 1 mM MgCl_2_, 35 mM NaCl, 0.5 mM EDTA, 0.5 mM DTT, 10% glycerol, 1 μg bovine serum albumin and 1.5 μg poly[d(I-C)] at room temperature for 30 min. Binding reactions were resolved in a 4% non-denaturing polyacrylamide gel (300 V, 90 min, 4 °C) in 0.5 × TBE. Gel was dried and exposed to X-ray film at −80 °C.

### Immunofluorescence microscopy

Cells were seeded on coverslips coated with poly-L-lysine (0.01%, Sigma), allowed to adhere overnight, and treated as described. After washing in PBS, cells were fixed with 4% paraformaldehyde for 20 min, permeabilized with 0.3% Triton X-100 in PBS for 5 min and blocked with PBS containing 10% FBS and 1% BSA for 1 h at room temperature. Cells were stained with primary antibody (anti-p65, sc-109, Santa Cruz Biotechnology) for 2 h, with secondary antibody (goat anti-rabbit-Alexa 488, Thermo Fisher) for 1 h and with DAPI (Sigma) for 15 min at room temperature. Between each step, cells were washed in PBS. Coverslips were mounted with ProLong Gold (Thermo Fisher) and imaged on a Stellaris confocal microscope (Leica) with a 63× oil-immersed objective. Three to six images per sample were acquired. Images were analyzed using Fiji software and nuclear p65 was quantified by measurement of green fluorescence intensity in the defined nuclear region [57].

### Statistical analysis and figure preparation

Statistical significance was determined by unpaired two-tailed Student’s *t* test for comparisons between two groups, or by 1- or 2-way ANOVA for multiple comparisons, followed by Sidak’s or Tukey post-hoc test. Differences were considered significant when *p* < 0.05. All statistical analyses were conducted using Prism 8 software (GraphPad). Model in Fig. 8 was created using BioRender (https://biorender.com/).

### Reporting summary

Further information on research design is available in the Nature Research Reporting Summary linked to this article.

## Supplementary information

supplementary

Reporting Summary

Original Data File

## Data Availability

All data generated and analyzed during this study are included in this article.
