# Supplementary material for: Mitochondrial reactive oxygen is critical for IL-12/IL-18-induced IFN-γ production by CD4+ T cells and is regulated by Fas/FasL signaling
Source: Cell Death Dis. 2022 Jun 6;13(6):531. doi: 10.1038/s41419-022-04907-5 (PMC9170726; doi:10.1038/s41419-022-04907-5)
Supplement: Supplementary file 1 — supplementary [file 41419_2022_4907_MOESM1_ESM.pdf]

Supplementary Materials for

Mitochondrial reactive oxygen is critical for IL-12/IL-18-induced IFN-γ production by CD4^{+} T cells and is regulated by Fas/FasL interaction

Gorjana Rackov*, Parinaz Tavakoli Zaniani, Sara Colomo del Pino, Rahman Shokri, Jorge Monserrat, Melchor Alvarez-Mon, Carlos Martinez-A and Dimitrios Balomenos*

*Corresponding authors. Emails: dbalomenos@cnb.csic.es; rackov@bio.mx

This PDF file includes:

Figs S1 to S8

Table S1

Table S2

Supplementary Figure 1

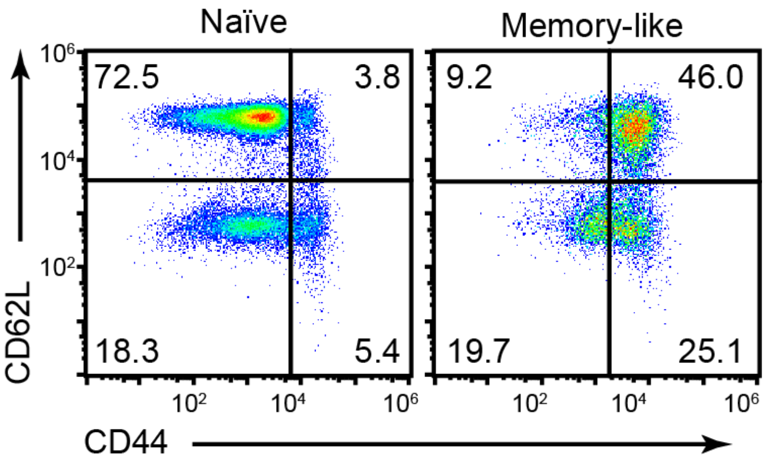

Supplementary Figure 1. Phenotyping of naïve and *in vitro* differentiated memory-like CD4$^+$ T cells. Naïve and in vitro differentiated memory-like CD4$^+$ T cells were analyzed by flow cytometry for surface expression of CD62L and CD44 markers. Pseudo-color plots are representative of three experiments performed.

Supplementary Figure 2

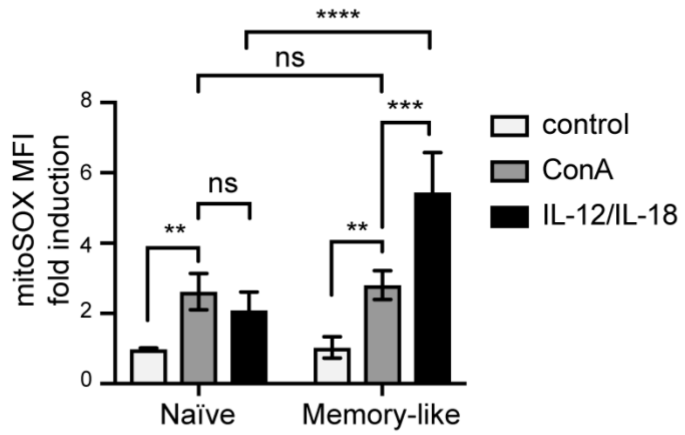

Supplementary Figure 2. mROS levels in naïve and memory-like CD4$^+$ T cells after stimulation. Flow cytometry analysis of mitoSOX red fluorescence showing MFI fold induction (over unstimulated cells) in naïve and memory-like cells at 1 hour after ConA or IL-12/IL-18 stimulation. The graphs show mean ± SD (*n* = 3 different mice), *$p<0.05$, **$p<0.01$, ***$p<0.001$, ****$p<0.0001$, 2-way ANOVA (with Sidak's correction for multiple comparison).

Supplementary Figure 3

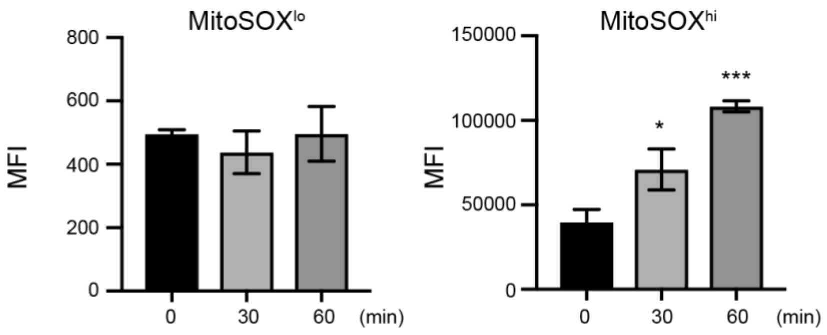

Supplementary Figure 3. mROS induction at early time points after stimulation. Flow cytometry analysis of mitoSOX red fluorescence showing the MFI within mitoSOX^{lo} and mitoSOX^{hi} populations at early time points after IL-12/IL-18 activation. Graphs show mean ± SD (n = 3 different mice); *p<0.05; ***p<0.001, one-way ANOVA with post-hoc Tukey test.

Supplementary Figure 4

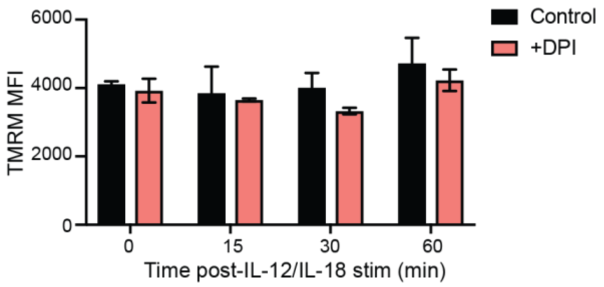

Supplementary Figure 4. DPI treatment did not affect mitochondrial membrane potential at early time points post IL-12/IL-18 treatment, measured flow cytometry of TMRM fluorescence. FCCP was used to depolarize the membrane and show background staining (not shown).

Supplementary Figure 5

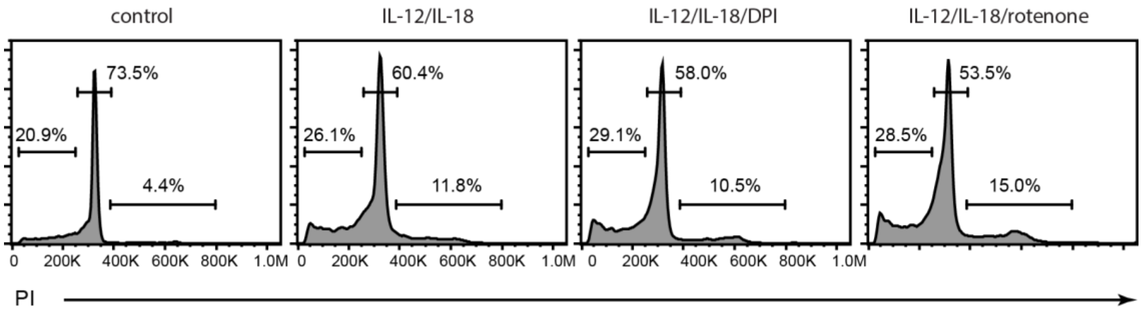

Supplementary Figure 5. Cell cycle analysis. $CD4^+$ T cells from mouse spleens were stimulated with ConA for 24 h, expanded in the presence of IL-2 for 6 days, and stimulated with IL-12/IL-18. After 24 h, cell cycle was analyzed by flow cytometry. DPI or rotenone treatment did not affect cell cycle compared with IL-12/IL-18-treated cells. Shown are representative data of 2 experiments performed.

Supplementary Figure 6

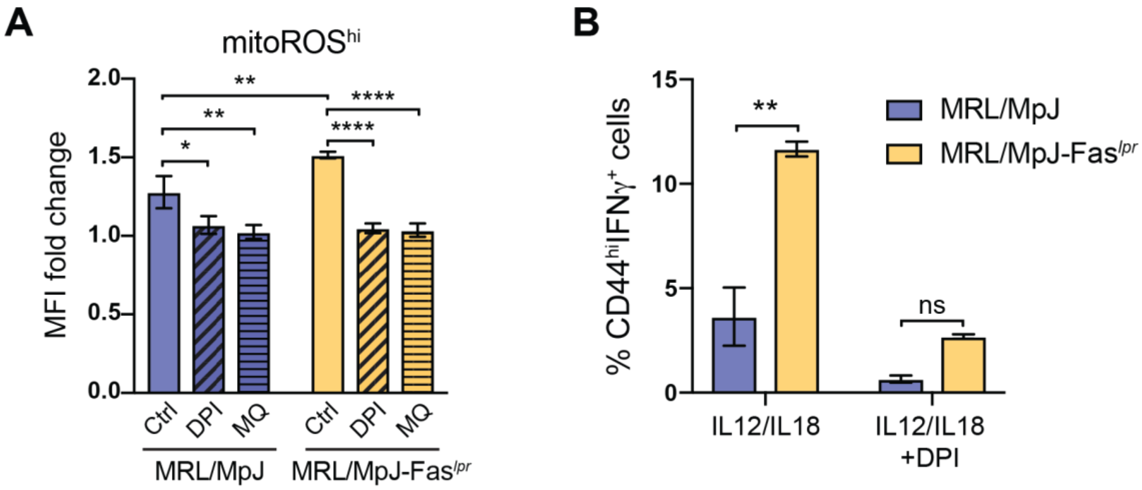

Supplementary Figure 6. Increased mROS and IFN-γ production in MRL/MpJ-Fas*lpr* memory-like CD4+ T cells compared with parental cells. In vitro differentiated memory-like CD4+ T cells from MRL/MpJ and MRL/MpJ-Fas*lpr* mice were stimulated with IL-12/IL-18. **(A)** Flow cytometry analysis of mitoROS fluorescence at 15 min after treatment with IL-12/IL-18 in presence of DPI or MitoQ. The samples were normalized to unstimulated cells of each treatment condition. **(B)** The frequency of IFN-γ-producing CD44hi cells 24h after IL-12/IL-18 or IL-12/IL-18 + DPI treatment, as detected by intracellular staining. Graphs show mean ± SD (*n*=3 different mice); $*p<0.05$; $**p<0.01$; $****p<0.0001$; ns, not significant; two-way ANOVA with Sidak's correction.

Supplementary Figure 7

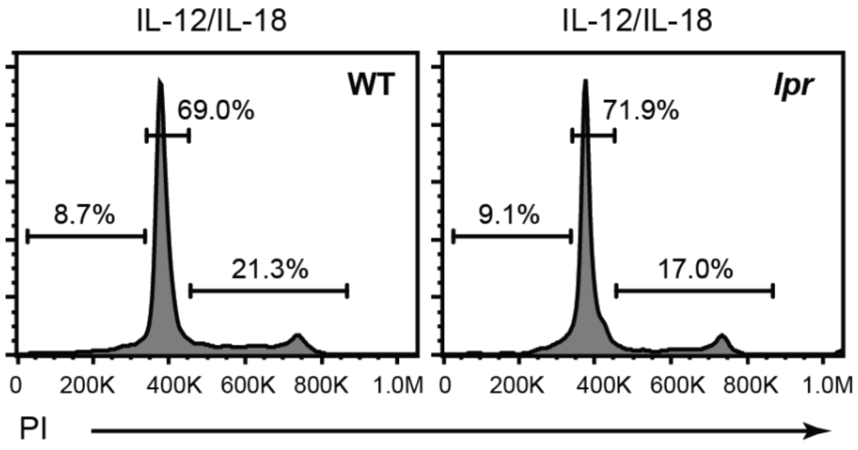

Supplementary Figure 7. Similar cell cycle profile in WT and *lpr* memory-like cells after stimulation with IL-12/IL-18. In vitro differentiated memory-like $CD4^+$ T cells from WT and *lpr* mice were stimulated with IL-12/IL-18 for 24 h. WT and *lpr* cells had similar cell cycle profiles with no apoptotic peak after IL-12/IL-18 stimulation. Shown are representative data of 2 experiments performed.

Supplementary Figure 8

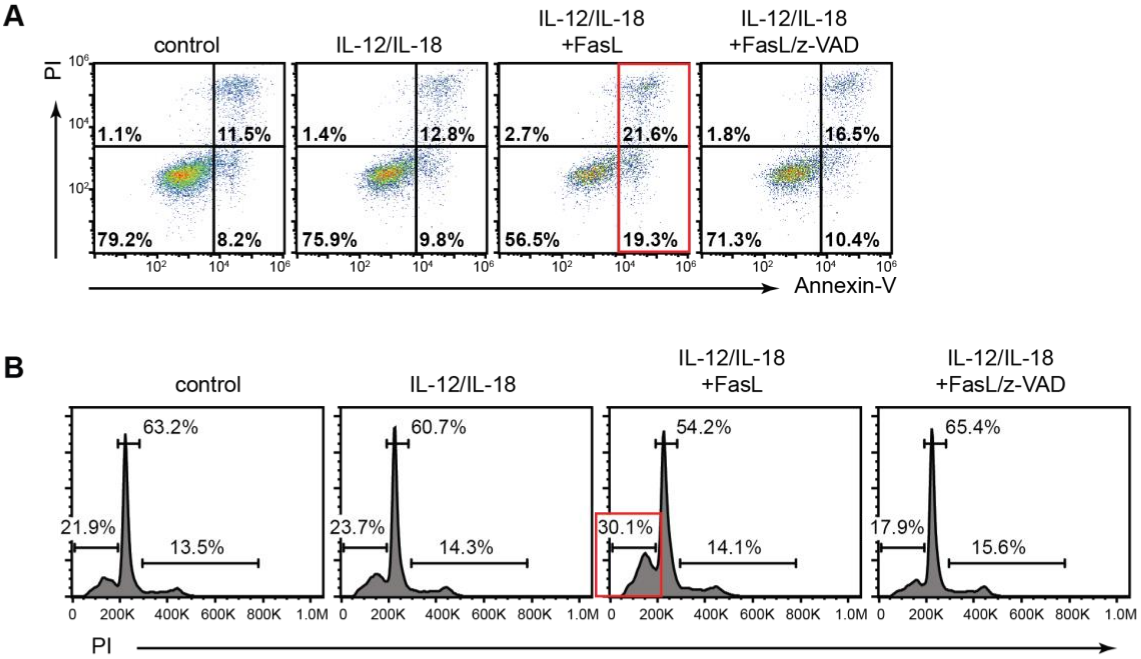

Supplementary Figure 8. Soluble FasL treatment induced apoptosis in IL-12/IL-18 stimulated cells, which was prevented by zVAD treatment. In vitro differentiated WT memory-like $CD4^+$ T cells were stimulated with IL-12/IL-18 for 4 h in presence of soluble FasL and crosslinker. zVAD was added to inhibit apoptosis. (**A**) Apoptosis was analyzed by flow cytometry after staining with AnnexinV-FITC and PI. Shown are the proportions of live (Annexin V-negative and PI-negative), early apoptotic (Annexin V-positive and PI-negative) and late apoptotic/necrotic (Annexin V-positive and PI-positive) cells (**B**) Cell cycle analysis showing apoptotic peak after stimulation with FasL, which was absent after treatment with zVAD. Shown are representative data of 2 experiments performed.

Table S1. siRNA duplexes

Gene name/ OriGene duplex name	Oligonucleotide sequence
Negative control SR30004	Universal scrambled negative control
Ndufaf1 SR410958A	rGrGrArUrGrGrUrArArArUrArUrCrArGrGrCrArArGrArCAC
Ndufaf1 SR410958B	rGrUrGrArCrUrUrCrUrGrArUrArArGrArCrArArUrUrGrGAG
Ndufaf1 SR410958C	rUrArCrUrUrArArGrArCrGrGrUrArArGrUrArCrArGrCrATG

All siRNAs were predesigned by OriGene.

Table S2. Primers for qRT-PCR

Gene name	Forward and reverse primer sequences (5’ => 3’)
Ifng	ATCTGGAGGAACTGGCAAAA<br>TTCAAGACTTCAAAGAGTCTGAGG
Csf2	GCATGTAGAGGCCATCAAAGA<br>CGGGTCTGCACACATGTTA
Il2	GCTGTTGATGGACCTACAGGA<br>TTCAATTCTGTGGCCTGCTT
Il4	CATCGGCATTTTGAACGAG<br>CGAGCTCACTCTCTGTGGTG
Ndufaf1	TGGGGACAGTAGACAAAGTGG<br>GACAGCTTCCTCTCAAAAGCAC
T-bet	CAACCAGCACCAGACAGAGA<br>ACAAACATCCTGTAATGGCTTG
Gata3	TTATCAAGCCCAAGCGAAG<br>TGGTGGTGGTCTGACAGTTC
