# Supplementary material for: Mitochondrial reactive oxygen is critical for IL-12/IL-18-induced IFN-γ production by CD4+ T cells and is regulated by Fas/FasL signaling
Source: Cell Death Dis. 2022 Jun 6;13(6):531. doi: 10.1038/s41419-022-04907-5 (PMC9170726; doi:10.1038/s41419-022-04907-5)

Full unedited gels

Figure 4A

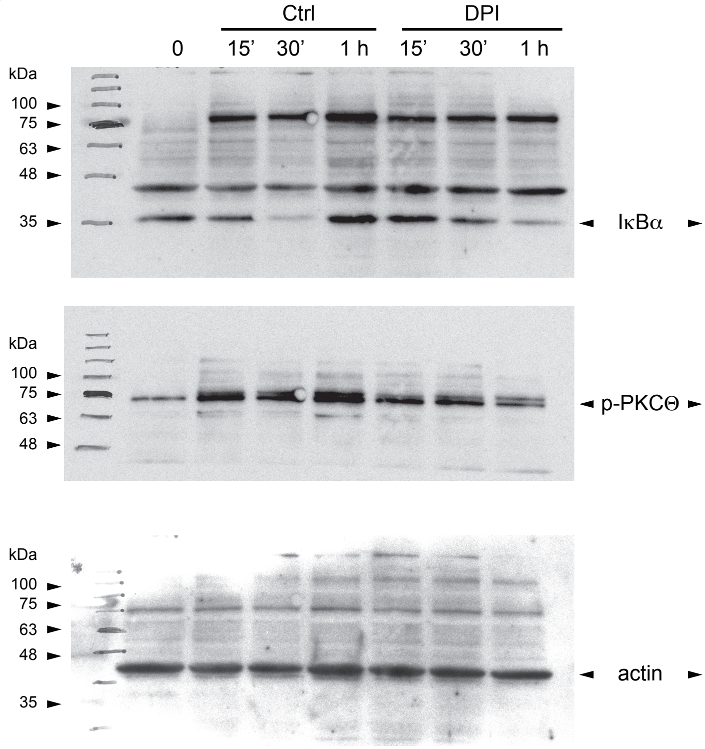

Figure 4B

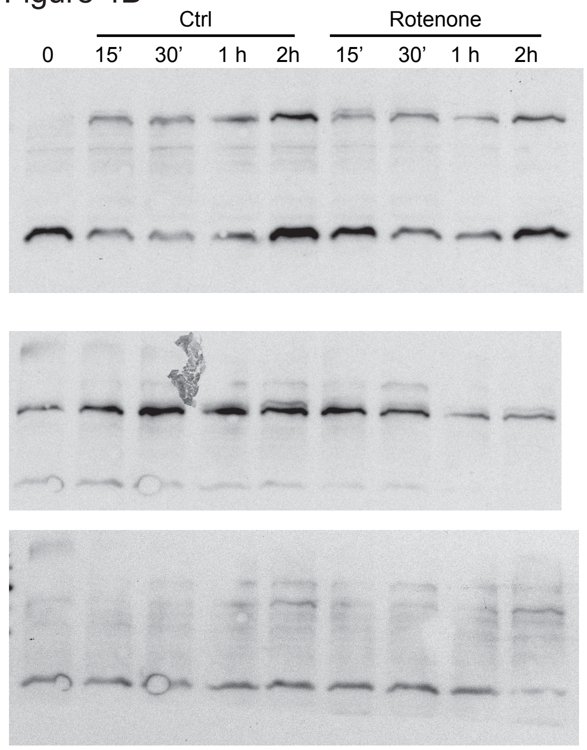

Figure 4F

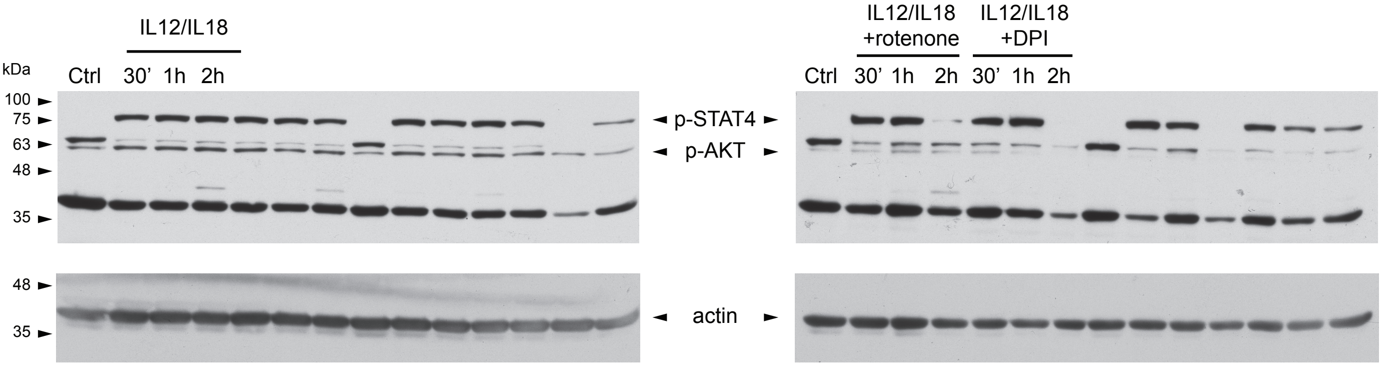

Figure 4G

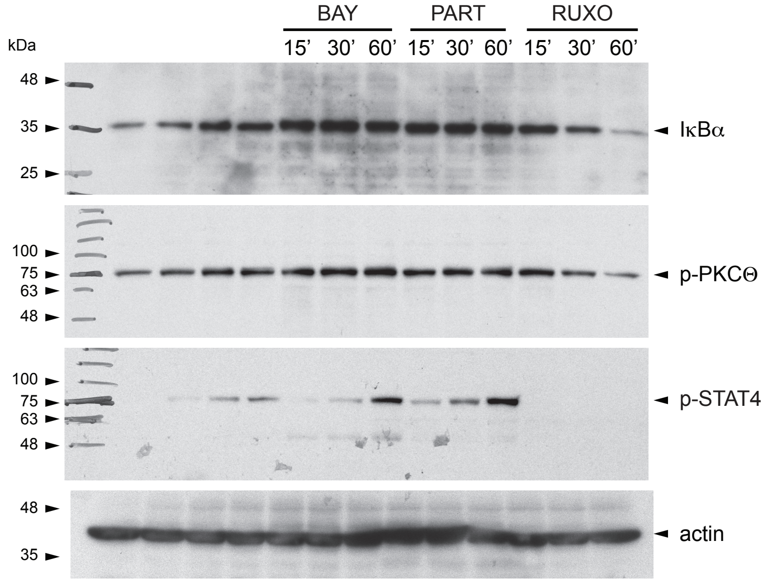

Figure 5B

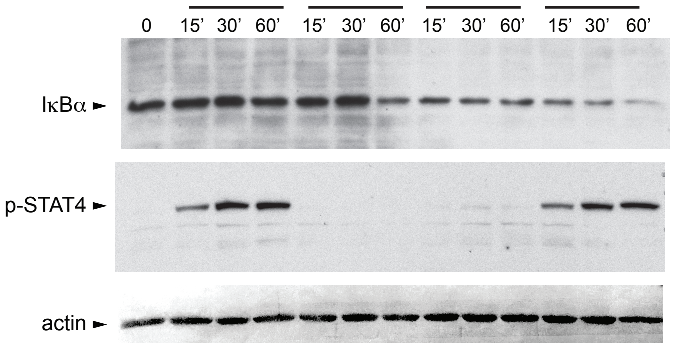

Full unedited gels

Figure 7A

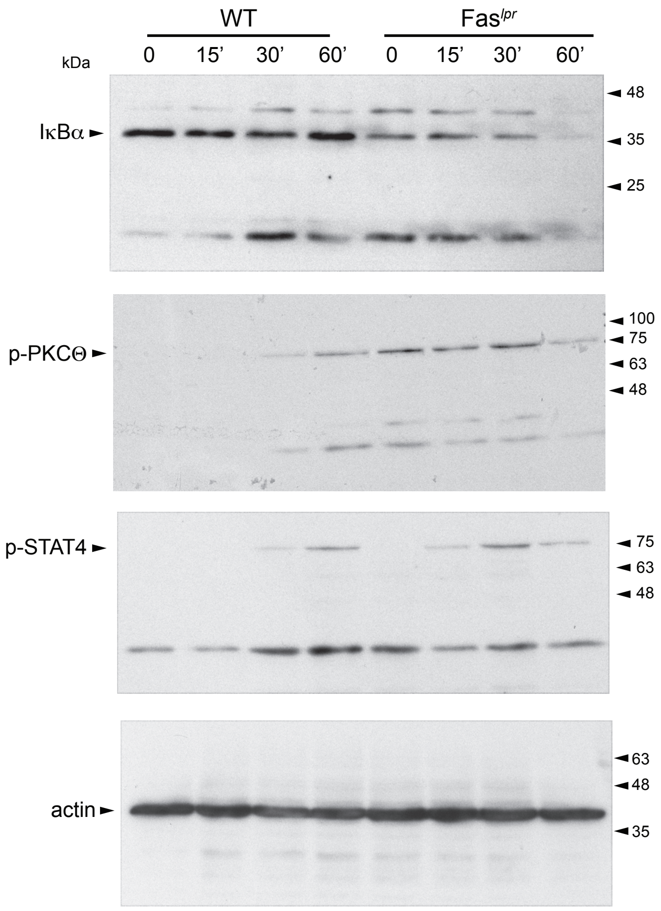

Figure 7G

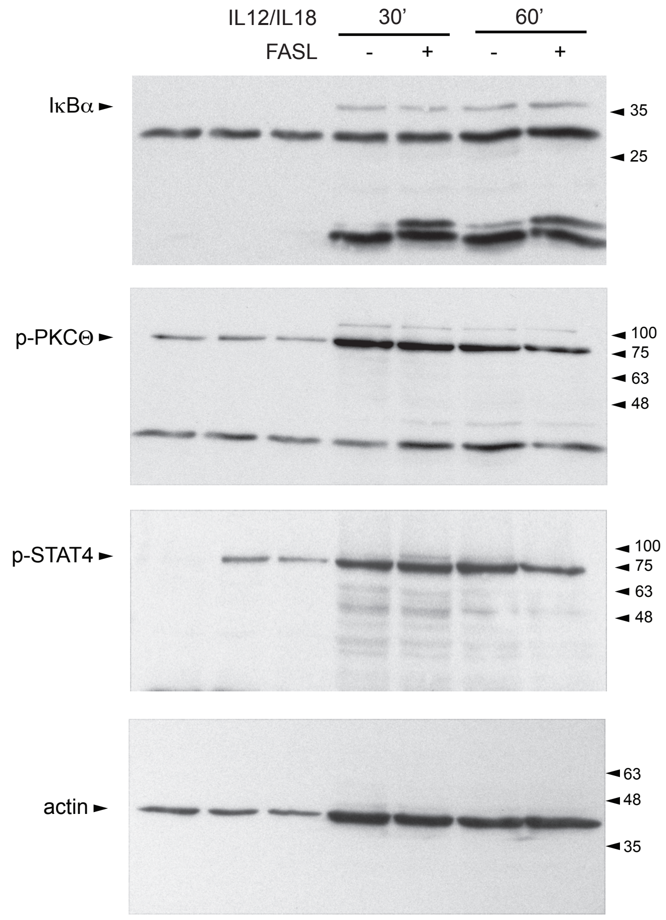

Figure 7H Left

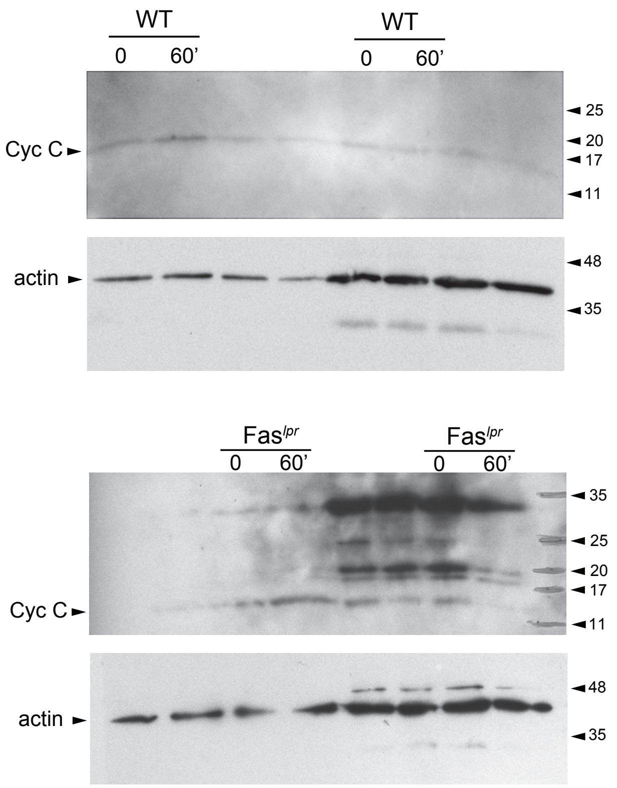

Figure 7H Right

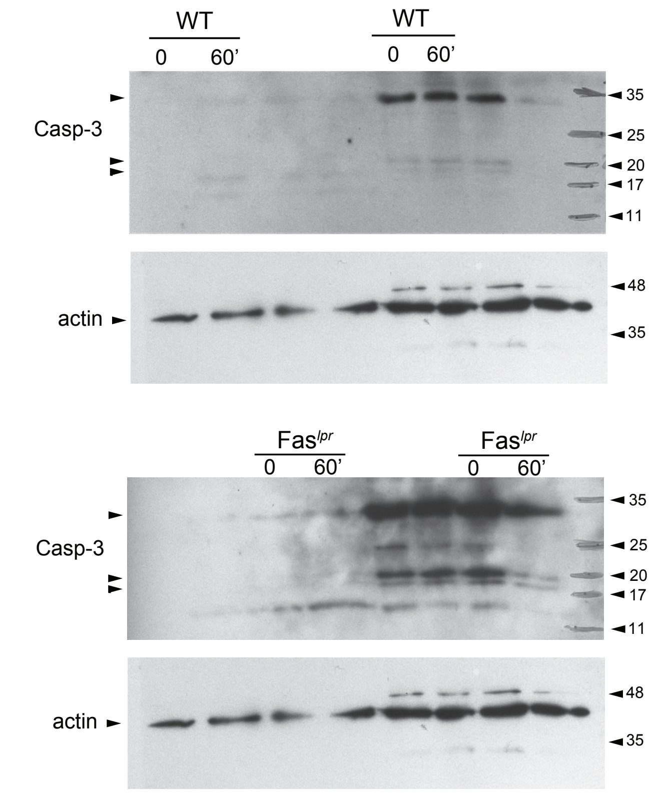

Supplement: Supplementary file 3 — Original Data File [file 41419_2022_4907_MOESM3_ESM.pdf]
